# Supplementary material for: Wastewater surveillance reveals patterns of antibiotic resistance across the United States
Source: medRxiv. 2025 Apr 4:2025.04.02.25325128. Preprint. [Version 1] doi: 10.1101/2025.04.02.25325128 (PMC11998803; doi:10.1101/2025.04.02.25325128)
Supplement: Supplement 1 [file media-1.pdf]

Supplementary Information

Wastewater surveillance reveals patterns of antibiotic resistance across the United States

Authors: Sooyeol Kim[1], Alessandro Zulli[2], Elana M. G. Chan[2], Dorothea Duong[3], Rebecca Y. Linfield[4], Caroline McCormack[1], Bradley J. White[3], Marlene K. Wolfe[5], Alexandria B. Boehm[2], Amy J. Pickering[1,6,7]

Corresponding author: pickering@berkeley.edu

Affiliation
[1]Department of Civil and Environmental Engineering, University of California, Berkeley, CA, 94720, USA
[2]Department of Civil and Environmental Engineering, Stanford University, Stanford, CA, 94305, USA
[3]Verily Life Sciences, South San Francisco, CA, 94080, USA
[4]Division of Infectious Diseases and Geographic Medicine, Department of Medicine, Stanford University, Stanford, CA, 94305, USA
[5]Gangarosa Department of Environmental Health, Rollins School of Public Health, Emory University, Atlanta, GA, 30329, USA
[6]Chan Zuckerberg Biohub San Francisco, San Francisco, California
[7]Blum Center for Developing Economies, University of California

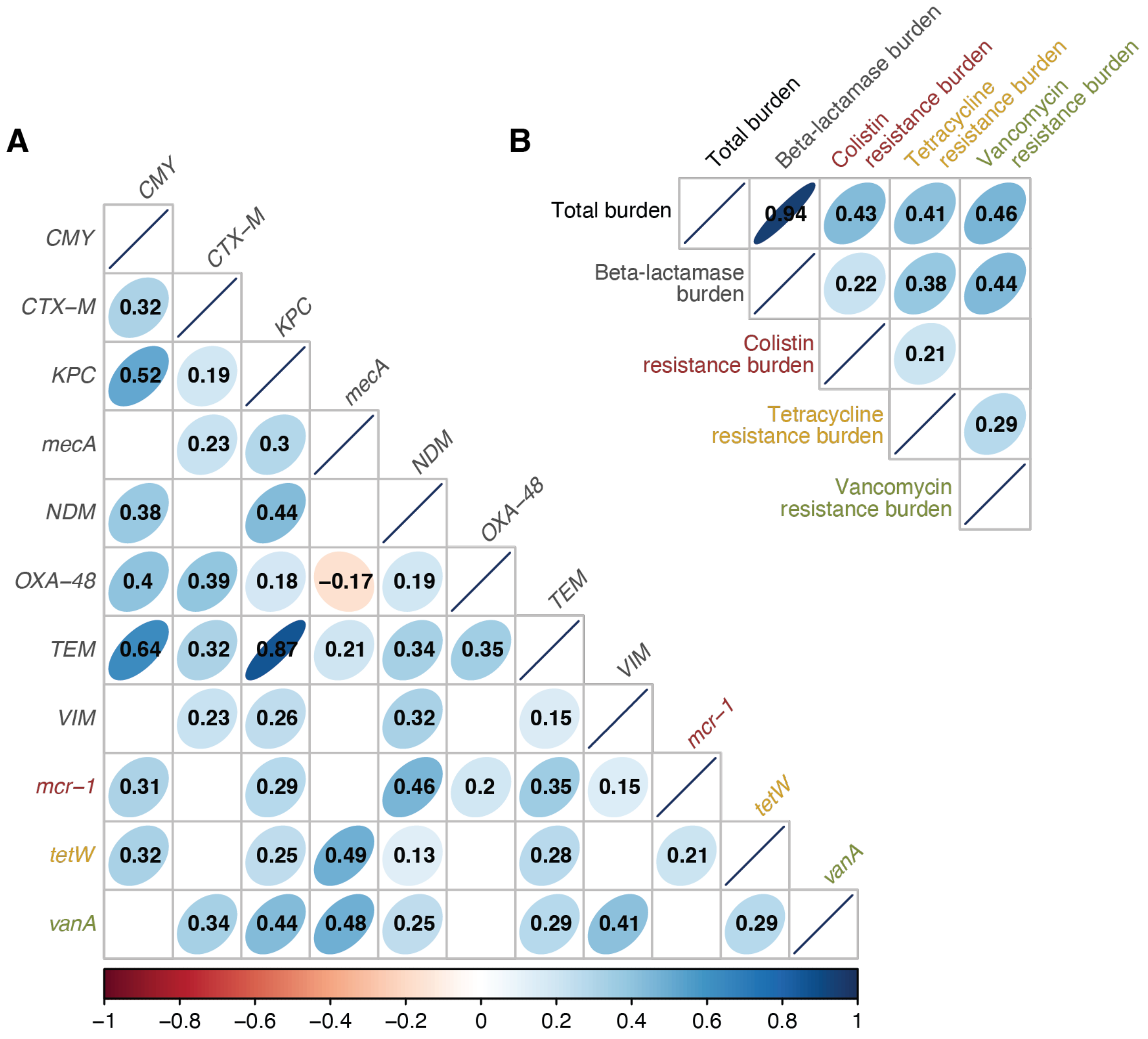

Figure S1. **Correlation among ARGs measured in wastewater. A)** Spearman's correlation coefficient among ARG concentrations normalized by 16S rRNA gene ($p < 0.05$). **B)** Spearman's correlation coefficient among antibiotic resistance burden calculated as z-scores based on distribution of each gene ($p < 0.05$).

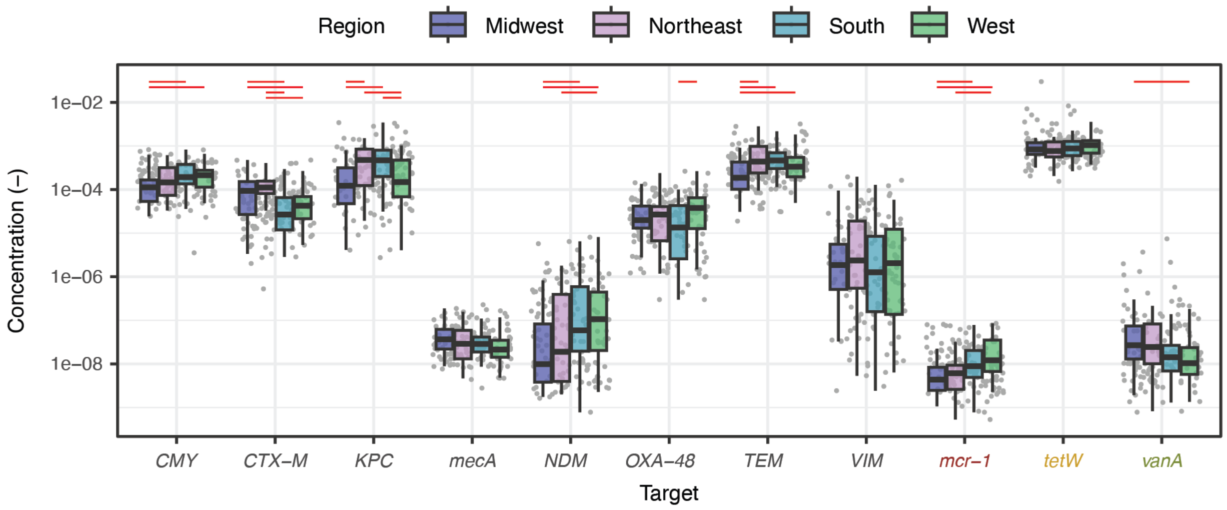

Figure S2. Regional distribution of all ARGs. Each data point represents a wastewater treatment plant. If the gene was undetected, half of the theoretical measurement limit was substituted as the measured value. The median is shown by the line inside the box with the 25th and 75th percentile represented by the lower and upper boundary of the box. Bottom and top whiskers show 1.5 x interquartile range. Red lines indicate a significant pairwise difference between regions measured by the Conover-Iman post-hoc test with Benjamini-Hochberg correction applied ($p < 0.05$).

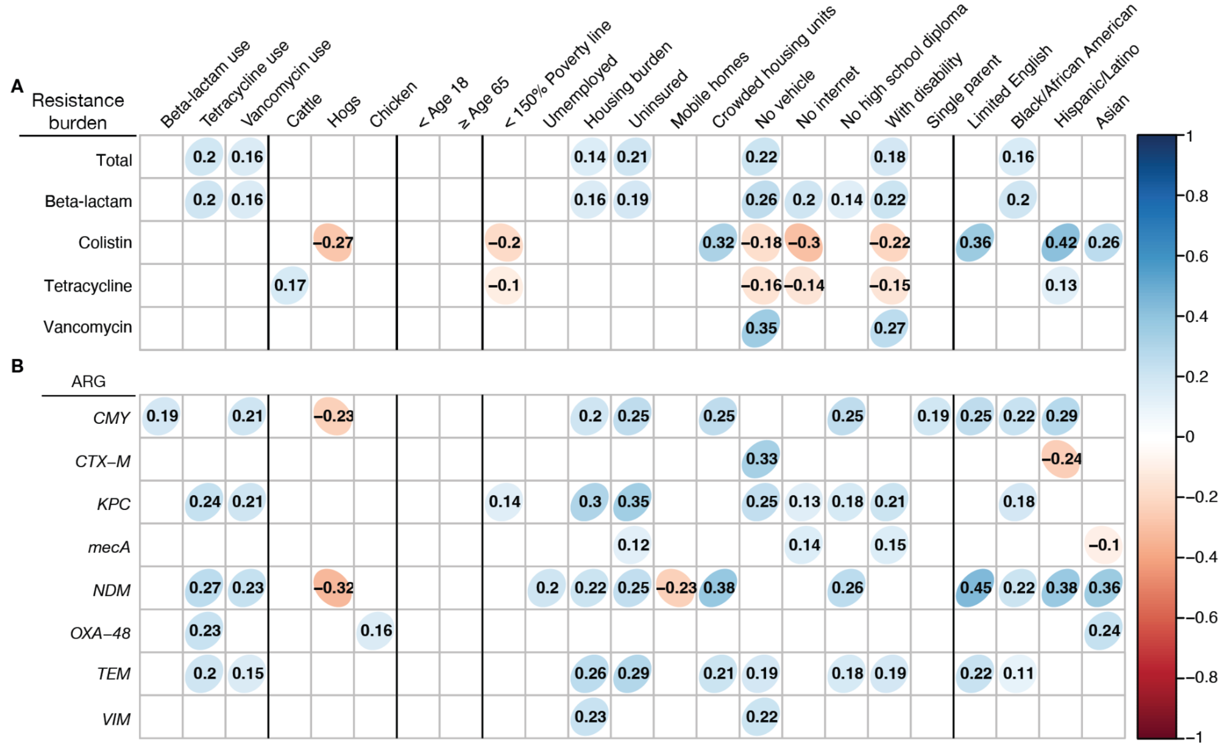

Figure S3. Unadjusted correlation resistance measured in wastewater and potential determinants. Spearman correlation coefficient among potential determinants of resistance burden and A) antibiotic resistance burden score and B) beta-lactamase gene concentrations measured in wastewater and normalized by 16S rRNA gene ($p < 0.05$).

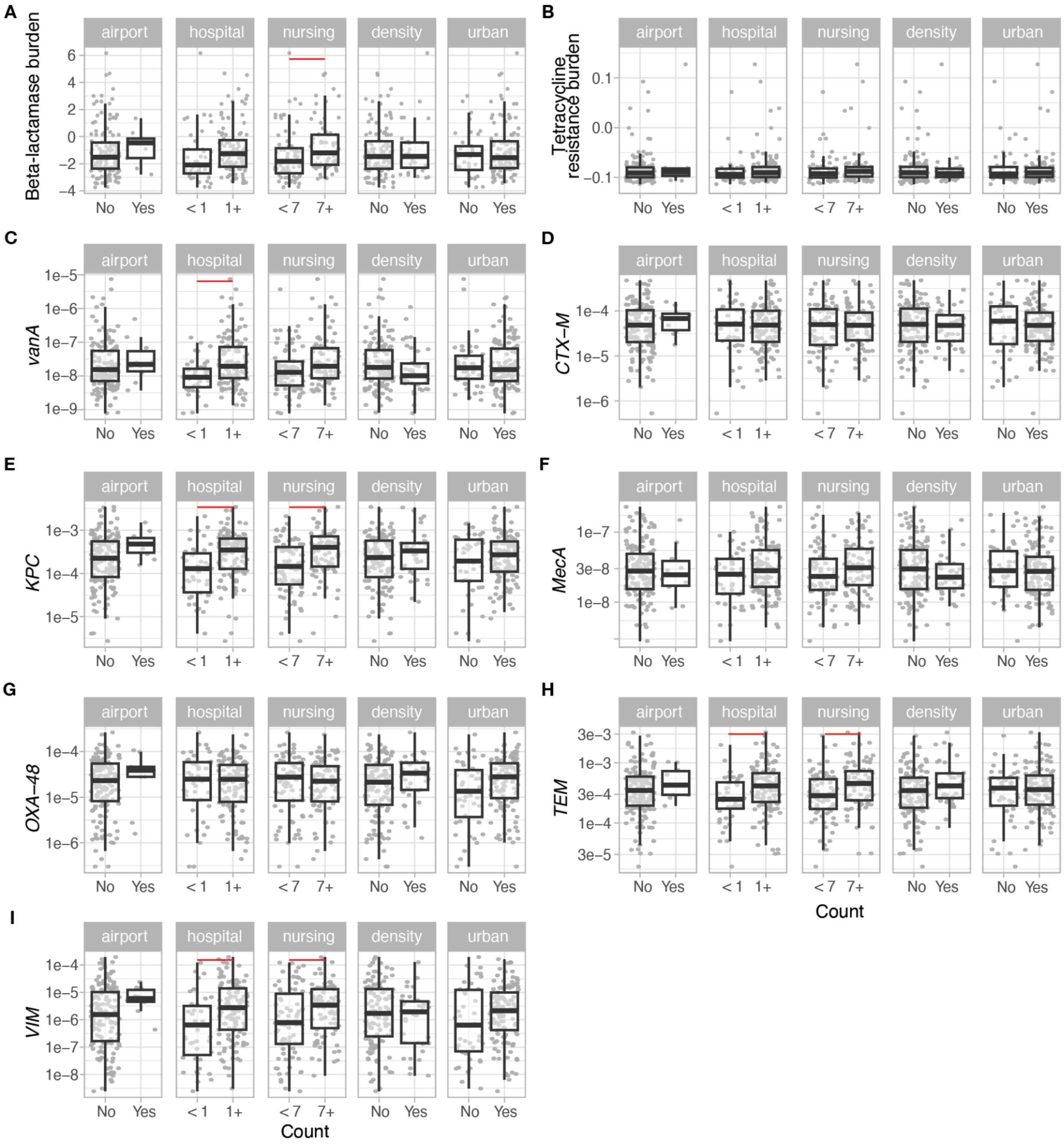

Figure S4. Comparison of categorical variables for antibiotic resistance burden and genes. Bivariate analysis of points of interest, population density, and urbanicity for **A)** beta-lactamase resistance burden and **B)** tetracycline resistance burden measured in wastewater as aggregate z-scores. **C)** Vancomycin resistance burden is shown as *vanA* concentration normalized by 16S rRNA for ease of visualization. Same analysis done for wastewater concentration normalized by 16S rRNA gene for individual beta-lactamase genes, **D)** *CTX-M*, **E)** *KPC*, **F)** *mecA*, **G)** *OXA-48*, **H)** *TEM*, **I)** *VIM*. The median is shown by the line inside the box with the 25th and 75th percentile represented by the lower and upper boundary of the box. Bottom and top whiskers show 1.5 x interquartile range. Red lines indicate a significant pairwise

difference between regions measured by the Wilcoxon post-hoc test with Benjamini-Hochberg correction applied ($p < 0.05$).

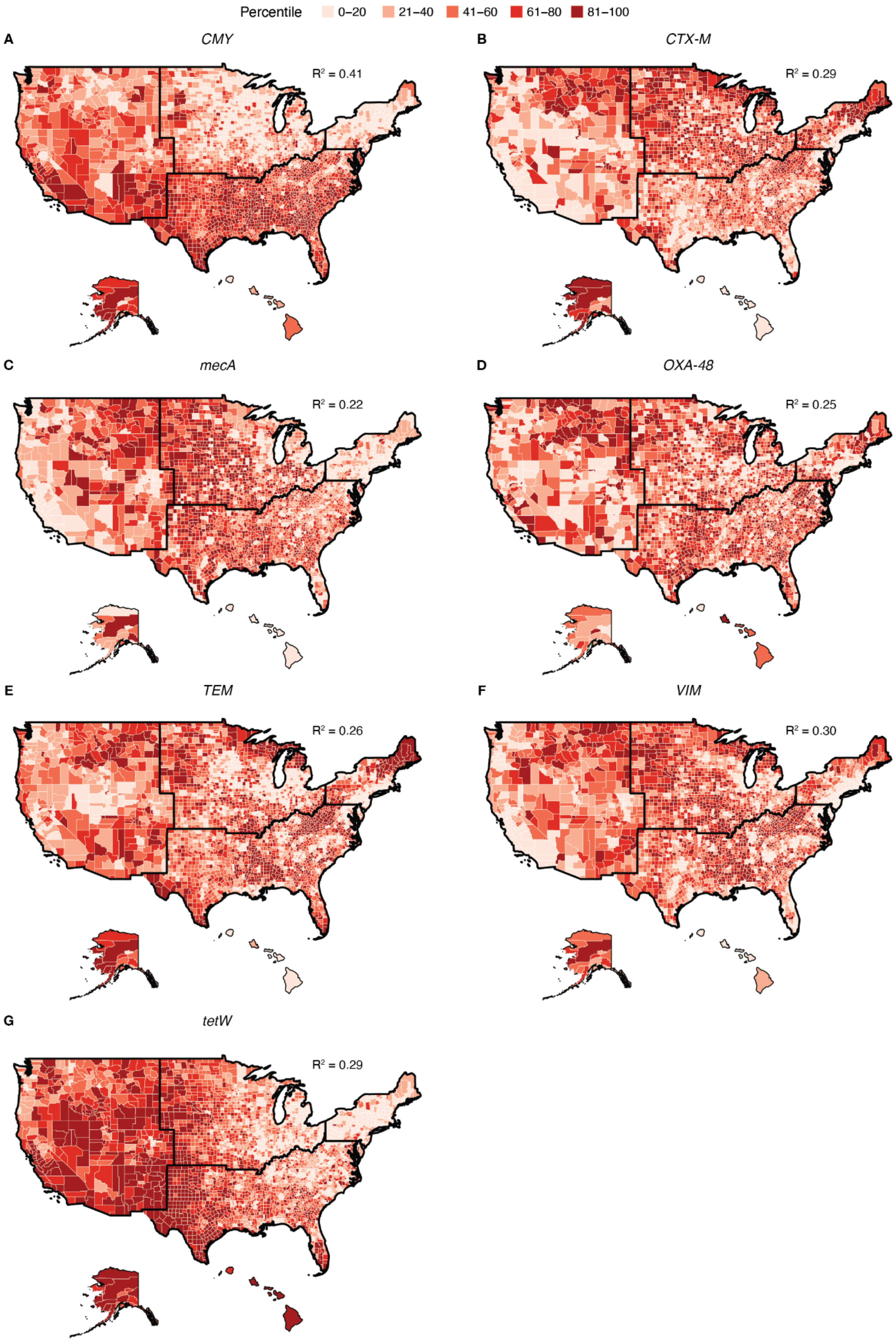

Percentile
0–20
21–40
41–60
61–80
81–100
A
CMY
R² = 0.41
B
CTX-M
R² = 0.29
C
mecA
R² = 0.22
D
OXA-48
R² = 0.25
E
TEM
R² = 0.26
F
VIM
R² = 0.30
G
tetW
R² = 0.29

Figure S5. Random forest modeling of antibiotic resistance gene concentrations across the United States using secondary data. Predicted **A)** CMY (R^2: 0.41), **B)** CTX-M (R^2: 0.29), **C)** mecA (R^2: 0.22), **D)** OXA-48 (R^2: 0.25), **E)** TEM (R^2: 0.26), **F)** VIM (R^2: 0.30), and **G)** tetW (R^2: 0.29) concentrations across the United States. The predicted concentration, normalized by 16S rRNA, is visualized as percentiles, with darker colors indicating higher concentrations. The random forest models are trained based on the secondary data in Table 1, with the exception of antibiotic prescription rates. A higher percentile indicates a higher predicted concentration within that county. Each map shows predictive performance of the model indicated by R^2. Alaska and Hawaii are not to scale.

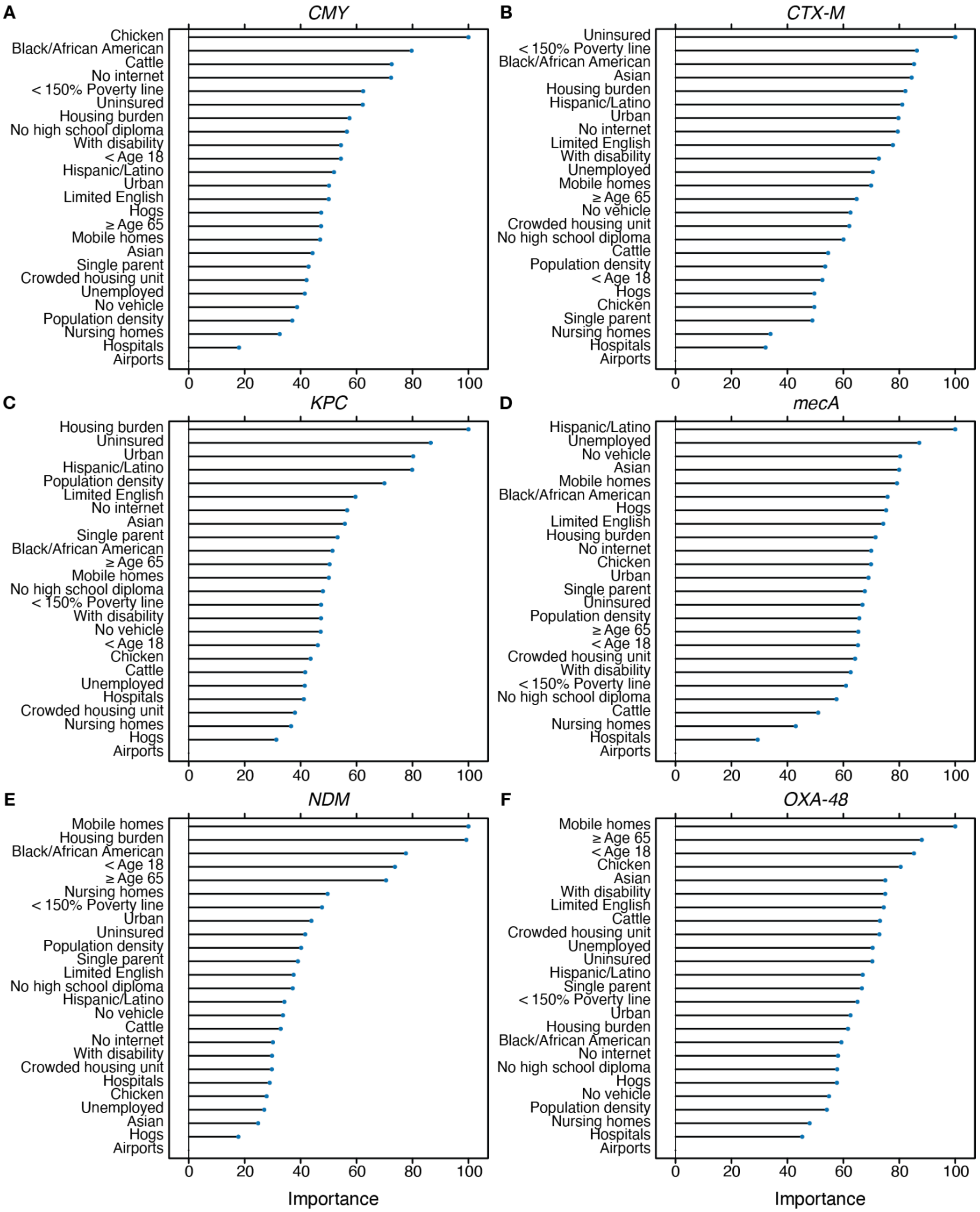
A
CMY
Chicken
Black/African American
Cattle
No internet
< 150% Poverty line
Uninsured
Housing burden
No high school diploma
With disability
< Age 18
Hispanic/Latino
Urban
Limited English
Hogs
≥ Age 65
Mobile homes
Asian
Single parent
Crowded housing unit
Unemployed
No vehicle
Population density
Nursing homes
Hospitals
Airports
B
CTX-M
Uninsured
< 150% Poverty line
Black/African American
Asian
Housing burden
Hispanic/Latino
Urban
No internet
Limited English
With disability
Unemployed
Mobile homes
≥ Age 65
No vehicle
Crowded housing unit
No high school diploma
Cattle
Population density
< Age 18
Hogs
Chicken
Single parent
Nursing homes
Hospitals
Airports
C
KPC
Housing burden
Uninsured
Urban
Hispanic/Latino
Population density
Limited English
No internet
Asian
Single parent
Black/African American
≥ Age 65
Mobile homes
No high school diploma
< 150% Poverty line
With disability
No vehicle
< Age 18
Chicken
Cattle
Unemployed
Hospitals
Crowded housing unit
Nursing homes
Hogs
Airports
D
mecA
Hispanic/Latino
Unemployed
No vehicle
Asian
Mobile homes
Black/African American
Hogs
Limited English
Housing burden
No internet
Chicken
Urban
Single parent
Uninsured
Population density
≥ Age 65
< Age 18
Crowded housing unit
With disability
< 150% Poverty line
No high school diploma
Cattle
Nursing homes
Hospitals
Airports
E
NDM
Mobile homes
Housing burden
Black/African American
< Age 18
≥ Age 65
Nursing homes
< 150% Poverty line
Urban
Uninsured
Population density
Single parent
Limited English
No high school diploma
Hispanic/Latino
No vehicle
Cattle
No internet
With disability
Crowded housing unit
Hospitals
Chicken
Unemployed
Asian
Hogs
Airports
F
OXA-48
Mobile homes
≥ Age 65
< Age 18
Chicken
Asian
With disability
Limited English
Cattle
Crowded housing unit
Unemployed
Uninsured
Hispanic/Latino
Single parent
< 150% Poverty line
Urban
Housing burden
Black/African American
No internet
No high school diploma
Hogs
No vehicle
Population density
Nursing homes
Hospitals
Airports
0 20 40 60 80 100
Importance

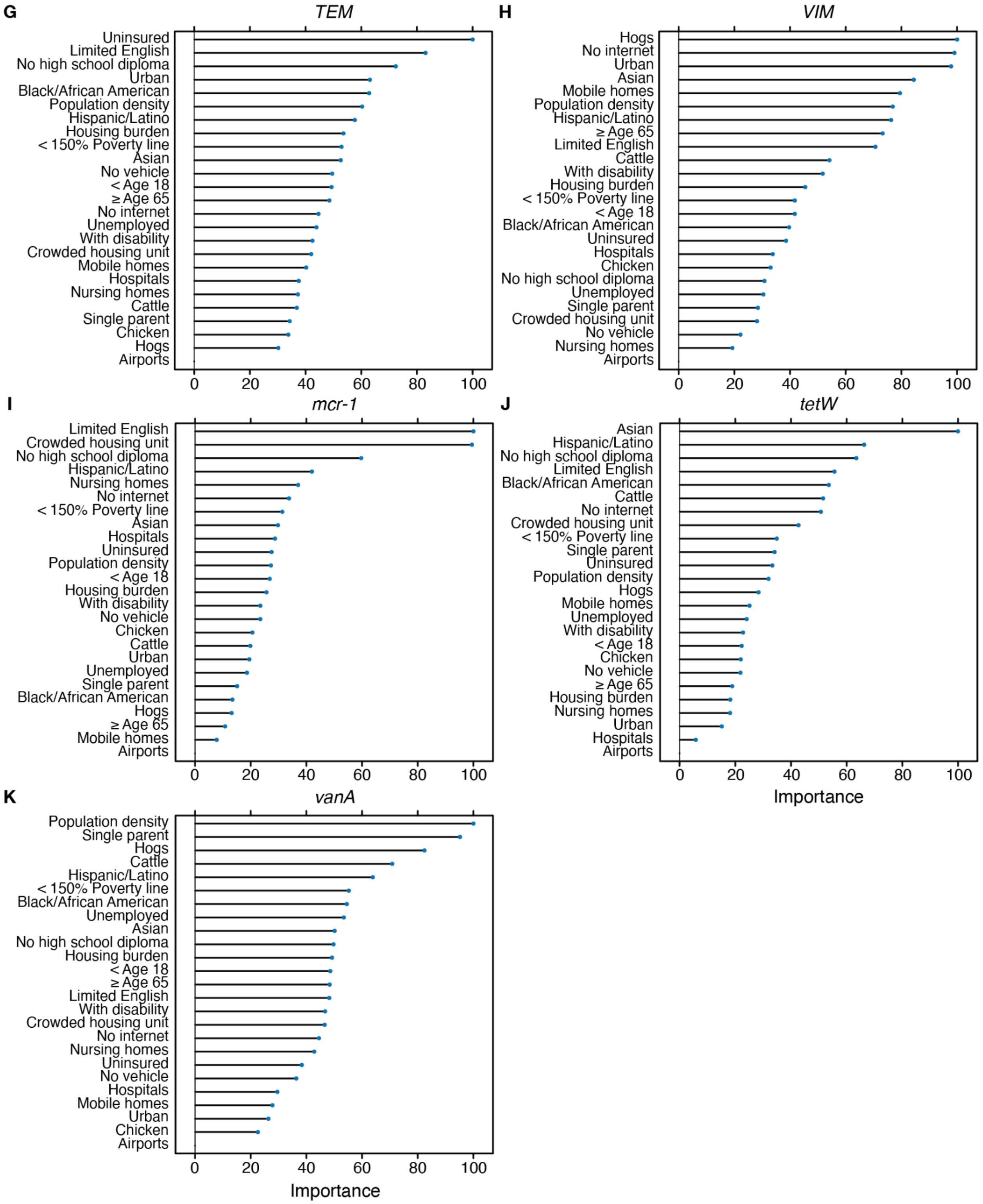

Figure S6. Variable importance for individual ARG random forest prediction model. Random forest model predicts relative concentration of ARGs normalized by 16S rRNA across all counties in the U.S. for **A)** *CMY*, **B)** *CTX-M*, **C)** *KPC*, **D)** *mecA*, **E)** *NDM*, **F)** *OXA-48*, **G)** *TEM*, **H)** *VIM*, **I)** *mcr-1*, **J)** *tetW*, and **K)** *vanA*.

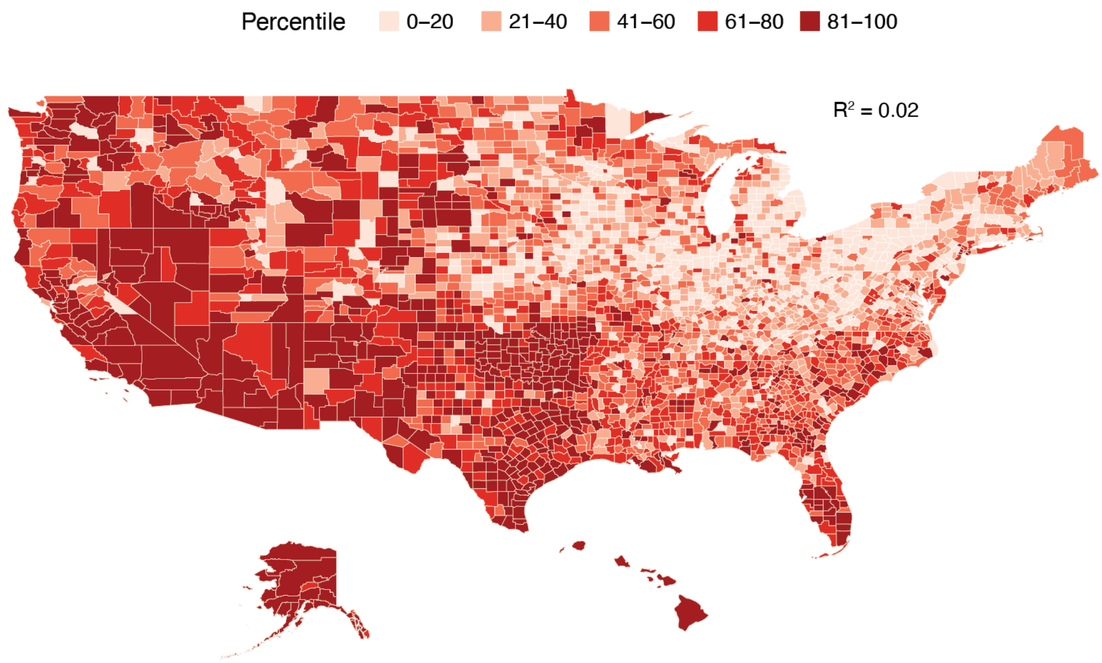

Figure S7. Random forest modeling of overall antibiotic resistance gene prevalence across the United States using secondary data. The predicted resistance gene prevalence using cumulative burden z-scores is visualized as percentiles, with darker colors indicating higher concentrations. The random forest models are trained based on the secondary data in Table 1, with the exception of antibiotic prescription rates. A higher percentile indicates a higher predicted concentration within that county. Predictive performance of the model indicated by R^2. Alaska and Hawaii are not to scale.

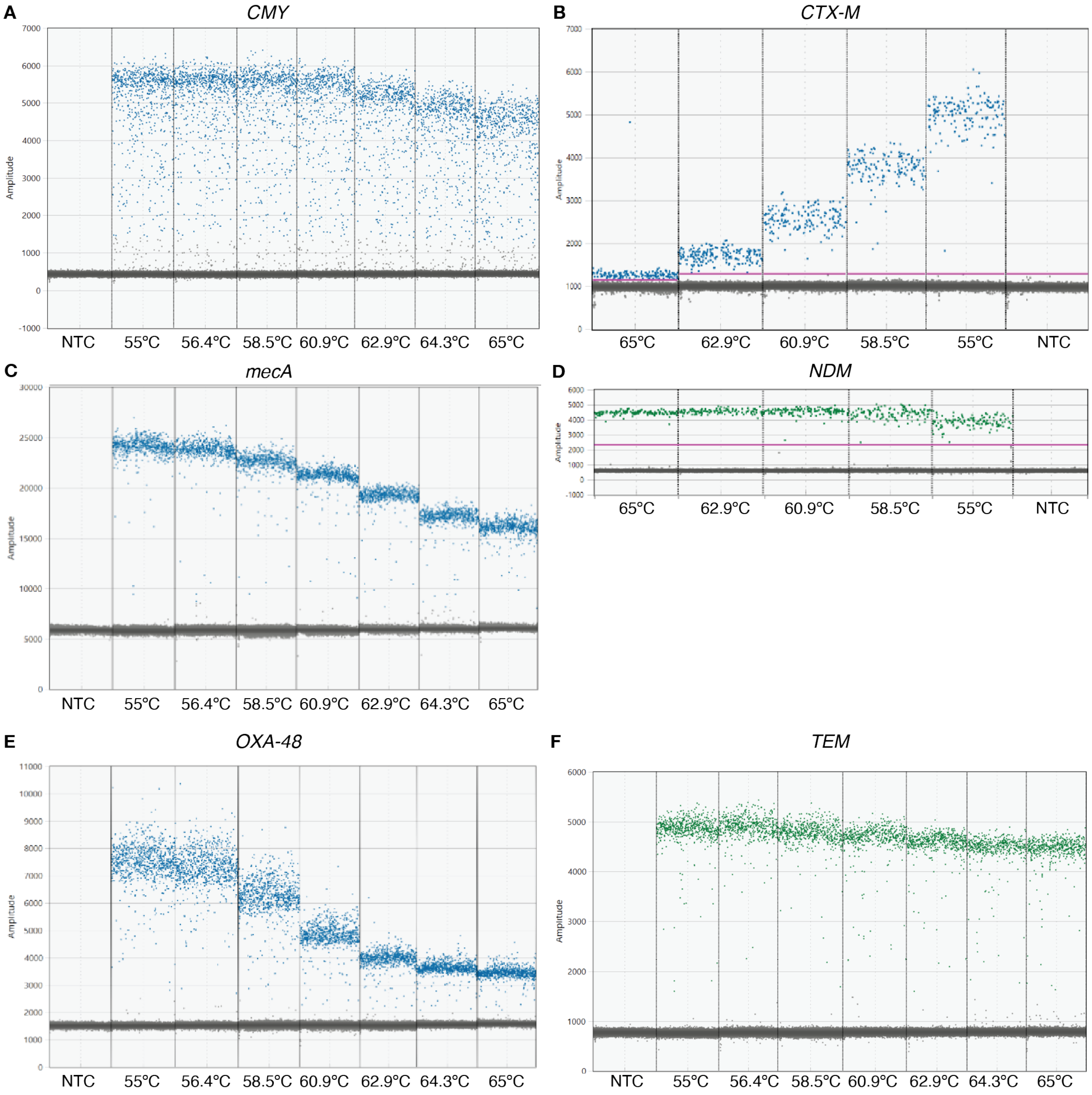
A
CMY
B
CTX-M
C
mecA
D
NDM
E
OXA-48
F
TEM
Amplitude
NTC
55°C
56.4°C
58.5°C
60.9°C
62.9°C
64.3°C
65°C

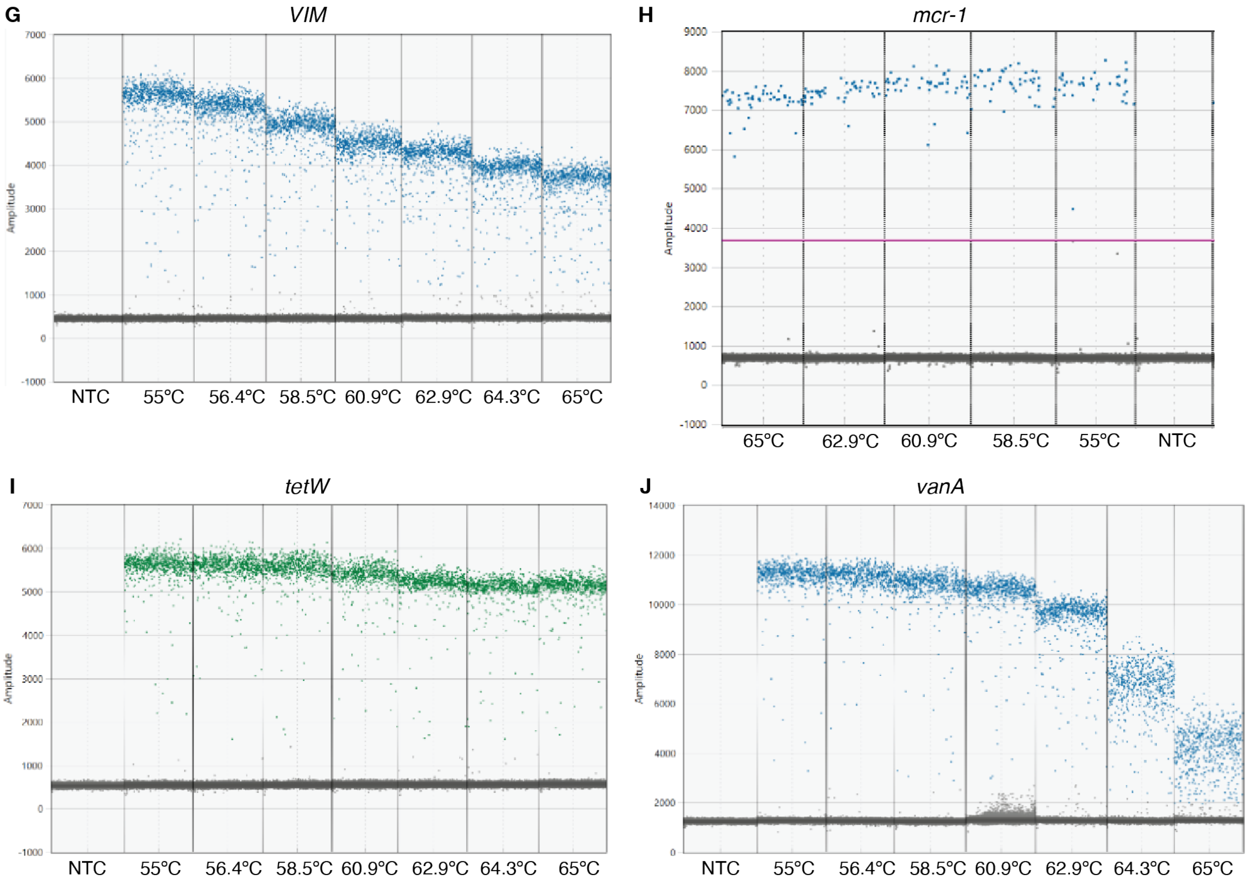

Figure S8. Temperature gradient test results for ARG assays. Screenshots for droplet digital PCR 1D plot of temperature gradient testing for **A)** *CMY*, **B)** *CTX-M*, **C)** *mecA*, **D)** *NDM*, **E)** *OXA-48*, **F)** *TEM*, **G)** *VIM*, **H)** *mcr-1*, **I)** *tetW*, and **J)** *vanA*. Temperature gradient testing was done with gblocks of target sequences (sequences provided in the SI), except for *NDM* and *CTX-M*, which were done with 1:10 and 1:100 diluted wastewater respectively.. Probes were either FAM (blue dots in the figure) or HEX (green dots in the figure). The pink line shows the automatic threshold by QX Manager. *KPC* was added later as the last target so instead of a temperature gradient testing, 58°C was tested to confirm that the assay performs well at the temperature set for other assays.

Table S1. **Summary of samples collected per census region.**

	All	Midwest	Northeast	South	West
**# of WWTPs**	163	32	24	48	59
**Total # of samples**	443	92	59	130	162
**Mean # of samples per WWTP**	2.7	2.9	2.5	2.7	2.7

Table S2. **Assays used in this study and expected concentration (high vs low) in wastewater samples from the U.S. based on preliminary testing of seven wastewater samples.**

Target	Sequences (5' - 3')	Expected Conc. (dilution)	Final Fluorophore	Amplicon Length	Ref.
CMY	F: AGACGTTTAACGGCGTGTTG<br>R: TAAGTGCAGCAGGCGGATAC<br>P: TATCGCCCGCGGCGAAAT	High (1:100)	Cy5	128	1
CTX-M	F: CCGTCACGCTGTTRTTAGGA<br>R: AATGCCACMCCCAGYCKKCC<br>P: CAGCAAAAACTTGCCGRATT	High (1:100)	ATTO590	109	2
KPC	F: GGCCGCCGTGCAATAC<br>R: GCCGCCCAACTCCTTCA<br>P:TGATAACGCCGCCGCCAATTTGT	High (1:100)	Cy5.5	61	3
mecA	F: CATTGATCGCAACGTTCAATTTAAT<br>R: TGGTCTTTCTGCATTCCTGGA<br>P: CTATGATCCCAATCTAACTTCCACATACC	Low (no dilution)	ATTO590	99	4
NDM	F: ATATCACCGTTGGGATCGAC<br>R: TAGTGCTCAGTGTCGGCATC<br>P: AAGGACAGCAAGGCCAAGTCG	Low (no dilution)	FAM	102	5
OXA-48	F: ACGGGCGAACCAAGCAT<br>R: GCGATCAAGCTATTGGGAATTT<br>P: TTACCCGCATCTACC	High (1:100)	HEX	60	6
TEM	F: GCATCTTACGGATGGCATGA<br>R: GTCCTCCGATCGTTGTCAGAA<br>P: CAGTGCTGCCATAACCATGAGTGA	High (1:100)	FAM	100	7
VIM	F: TSTACCCRTCCAATGGTCTC<br>R: AGAAGKGCCRCTGTGTTTTT<br>P:TGTCCGTGATGGYGATGAGTTG	Low (no dilution)	ROX	91	2
mcr-1	F: CATCGCGGACAATCTCGG<br>R: AAATCAACACAGGCTTTAGCAC<br>P: AACAGCGTGGTGATCAGTAGCAT	Low (no dilution)	HEX	116	8
tetW	F: GCAGAGCGTGGTTCAGTCT<br>R: GACACCGTCTGCTTGATGATAAT<br>P: TTCGGGATAAGCTCTCCGCCGA	High (1:100)	ROX	66	9

vanA	F: ATCAACCATGTTGATGTAGC<br>R: AAGGGATACCGGACAATTCA<br>P: TCCATCTTCACCTGACTTGCCA	Low (no dilution)	Cy5.5	94	[10]
16S	F: CGGTGAATACGTTCYCGG<br>R: GGWTACCTTGTTACGACTT	(1:50,000)	EvaGreen		[11]

All primers and probes ordered from IDT DNA (Maryland, USA).

Table S3. **Samples provided from each WWTP.** Tables of Locations for AMR Manuscript

gBlock sequences for assays

Assays were initially tested with gBlocks containing the target region and surrounding bases for each ARG assay. Maximum of three target regions were combined to create one gBlock as shown below, separated by a cluster of thymines.

For *TEM, NDM* (contains sequences for CTX-M corresponding to an assay not used in this study):
AGAGACACCACCACGCCGCGGGCGATGGCGCAGACGTTGCGTCAGCTTACGCTGGGTCA
TGCGCTGGGCGAAACCCAGCGGGCGCAGTTGGTGACGTGGCTCAAAGGCAATACGACCG
GCGCAGCCAGCATTCGGGCCGGCTtttttACTCACCAGTCACAGAAAAGCATCTTACGGATGG
CATGACAGTAAGAGAATTATGCAGTGCTGCCATAACCATGAGTGATAACACTGCGGCCAAC
TTACTTCTGACAACGATCGGAGGACCGAAGGAGCTAACCGtttttTCGATACCGCCTGGACCG
ATGACCAGACCGCCCAGATCCTCAACTGGATCAAGCAGGAGATCAACCTGCCGGTCGCGC
TGGCGGTGGTGACTCACGCGCATCgcggccgc

For *CMY, tetW, vanA*:
GGTCGGTCAGTAAGACGTTTAACGGCGTGTTGGGCGGCGACGCTATCGCCCGCGGCGAA
ATTAAGCTCAGCGATCCGGTCACGAAATACTGGCCAGAACTGACAGGCAAACAGTGGCGG
GGTATCAGCCTGCTGCACTTAGCCACCTATACAGCGtttttTGGCGTTGATTTGCAGAGCGTG
GTTCAGTCTGTTCGGGATAAGCTCTCCGCCGATATTATCATCAAGCAGACGGTGTCGCTGT
CCCCGGtttttATGCACGGATTACTTGTTAAAAAGAACCATGAATATGAAATCAACCATGTTGAT
GTAGCATTTTCAGCTTTGCATGGCAAGTCAGGTGAAGATGGATCCATACAAGGTCTGTTTG
AATTGTCCGGTATCCCTTTTGTAGGCTGCGATATTCAAAGCTCAGCAATTTGTATGGACAAg
cggccgc

For *mecA* (contains sequences for *mcr-1* and *SHV* corresponding to assays not used in this study):
TATCCCATCGCGGACAATCTCGGCTTTGTGCTGACGATCGCTGTCGTGCTCTTTGGCGCG
ATGCTACTGATCACCACGCTGTTATCATCGTATCGCTATGTGCTAAAGCCTGTGTTGATTTT
GCTATTAATCATGGGCGCGGTGACCAGTTATTTTACTGACACTTATGGCACtttttCTGGCGCG
CCGATGAACGCTTTCCCATGATGAGCACCTTTAAAGTAGTGCTCTGCGGCGCAGTGCTGG
CGCGGGTGGATGCCGGTGACGAACAGCTGGAGCGAAAGATCCACTATCGCCAGCAGGAT
CTGGTGGACTACTCGCCGGTCtttttTGCTCAATATAAAATTAAAACAAACTACGGTAACATTGA
TCGCAACGTTCAATTTAATTTTGTTAAAGAAGATGGTATGTGGAAGTTAGATTGGGATCATA

GCGTCATTATTCCAGGAATGCAGAAAGACCAAAGCATACATATTGAAAATTTAAAATgcggccg
c

For *OXA-48, VIM* (contains sequence for *IMP* corresponding to an assay not used in this study):
CAAGGATTTACCAATAATCTTAAACGGGCGAACCAAGCATTTTTACCCGCATCTACCTTTAA
AATTCCCAATAGCTTGATCGCCCTCGATTTGGGCGTGGTTAAGtttttGCAGTCGTTTGATGGC
GCGGTCTACCCGTCCAATGGTCTCATTGTCCGTGATGGTGATGAGTTGCTTTTGATTGATA
CAGCGTGGGGTGCGAAAAACACAGCGGCACTTCTCGCGGAGATTGAAAAGCAAATTtttttTC
CAGGGCACACTCCAGATAACGTAGTGGTTTGGCTACCTGAAAATAGAGTTTTGTTCGGTGG
TTGTTTTGTTAAACCGTACGGTCTTGGTAATTTGGGTGACGCAAATTTAGAAGCTTGGCCAA
AGTCCGCCAAATTATTAATGTCCgcggccgc

For *mcr-1* (contains sequences for *SHV* and *IMP* corresponding to assays not used in this study):
CCCGAATAACAAAGCAGAGCGCATTGTAGTGATTTATCTGCGGGATACCCCGGCGAGCAT
GGCCGAGCGAAATCAGCAAATCGCCGGGATCGGCGCGGCGCTGATCGAGCACTGGCAAC
GCTAAtttttCAGCACGGGCGGAATAGAGTGGCTTAATTCTCGATCTATCCCCACGTATGCATC
TGAATTAACAAATGAACTGCTTAAAAAAGACGGTAAGGTTCAAGCCACAAATTCATTTAGCG
GAGTTAACTATTGGCTAGTTAAAAATAAAATTGAAGTTTTTTATCCAGGCCCGGGACACACT
CCAGATAACGTAGTGGTTTGGTTGCCTGAAAGGtttttGCCAAACCTATCCCATCGCGGACAAT
CTCGGCTTTGTGCTGACGATCGCTGTCGTGCTCTTTGGCGCGATGCTACTGATCACCACG
CTGTTATCATCGTATCGCTATGTGCTAAAGCCTGTGTTGATTTTGCTATTAATCATGGgcggcc
gc

For *KPC* (contains sequence for *vanB* corresponding to an assay not used in this study):
GACGGTGGCGGAGCTGTCCGCGGCCGCCGTGCAATACAGTGATAACGCCGCCGCCAATT
TGTTGCTGAAGGAGTTGGGCGGCCCGGCCGGGCTGACGGCCtttttGGGGAACGAGGATGA
TTTGATTGTCGGCGAAGTGGATCAAATCCGGCTGAGCCACGGTATCTTCCGCATCCATCAG
GAAAACGAGCCGGAAAAAGGCgcggccgc

Preparation of secondary data

Demographic and socioeconomic factors. The 2022 Social Vulnerability Index (SVI) from the US CDC includes various demographic and socioeconomic factors from the 2022 5-year American Community Survey (ACS) that relate to a community's social vulnerability or ability to respond to external hazards.[12] For each sewershed, we approximated the value of selected variables used in the SVI as a proportion using the relevant ACS variables obtained at the census tract resolution (**Table S4**).[13] Census tract and sewershed boundaries did not align, so we first aggregated ACS variable counts across all census tracts intersecting a sewershed to estimate counts for each sewershed. Specifically, we used the Tabulate Intersection geoprocessing tool in ArcGIS Pro (version 3.1.1) to determine the area proportion p of each intersecting census tract n in the sewershed.[14] Next, we adjusted the census tract-level count of each ACS variable based on p (**Equation 1**). Then we summed the adjusted counts across all N

census tracts intersecting the sewershed to determine the sewershed-level count for each ACS variable (**Equation 2**). Using sewershed-level counts of each ACS variable, we calculated the sewershed-level value of each SVI variable as a proportion as described in **Table S4**. The distribution of each proportion among sewersheds in the study is shown in **Figure S9**.

Equation 1. $count\_adjusted_n = count_n \times p_n$
Equation 2. $count\_sewershed \ = \sum_{n=1}^{N} \quad count\_adjusted_n$

Table S4. Selected main and adjunct variables in the Social Vulnerability Index (SVI) calculated as proportions using relevant American Community Survey (ACS) variables

Proportion	Description	Calculation using ACS variables[a]
< 150% Poverty line	Proportion of the population for whom poverty status is determined below the 150% poverty line	S1701_C01_040E / S1701_C01_001E
Unemployed	Proportion of the civilian labor force age 16+ years that is unemployed	DP03_0005E / DP03_0003E
Housing burden	Proportion of occupied housing units that are housing cost-burdened with an annual income <$75K	(S2503_C01_028E + S2503_C01_032E + S2503_C01_036E + S2503_C01_040E) / S2503_C01_001E
No high school diploma	Proportion the population age 25+ years with no high school diploma	B06009_002E / B06009_001E
Uninsured	Proportion of the civilian noninstitutionalized population that is uninsured	S2701_C04_001E / S2701_C01_001E
≥ Age 65	Proportion of the population age 65+ years	S0101_C01_030E / S0601_C01_001E
< Age 18	Proportion of the population age 17 years and younger	DP05_0019E / S0601_C01_001E
With disability	Proportion of the civilian noninstitutionalized population with a disability	DP02_0072E / S2701_C01_001E
Single parent	Proportion of single-parent households with children <18 years	(DP02_0007E + DP02_0011E) / (DP02_0006E + DP02_0010E)
Limited English	Proportion of persons age 5+ years who speak English "less than well"	(B16005_007E + B16005_008E + B16005_012E + B16005_013E + B16005_017E + B16005_018E + B16005_022E + B16005_023E + B16005_029E + B16005_030E + B16005_034E + B16005_035E + B16005_039E + B16005_040E + B16005_044E + B16005_045E) / B16005_001E
Black/African American	Proportion of the population that is Black/African American, not Hispanic or Latino	DP05_0080E / S0601_C01_001E
Hispanic/Latino	Proportion of the population this is Hispanic or Latino	DP05_0073E / S0601_C01_001E
Asian	Proportion of the population that is Asian, not Hispanic or Latino	DP05_0082E / S0601_C01_001E
Mobile homes	Proportion of housing units that are mobile homes	DP04_0014E / DP04_0001E
Crowded housing units	Proportion of occupied housing units with more people than rooms	(DP04_0078E + DP04_0079E) / DP04_0002E

No vehicle	Proportion of occupied housing units with no vehicle	DP04_0058E / DP04_0002E
No internet	Proportion of households with no internet	S2801_C01_019E / DP02_0001E

[a] Variables are from the 2022 5-year American Community Survey (ACS). Census tract-level counts for each variable were first adjusted and summed by sewershed to obtain sewershed-level counts.

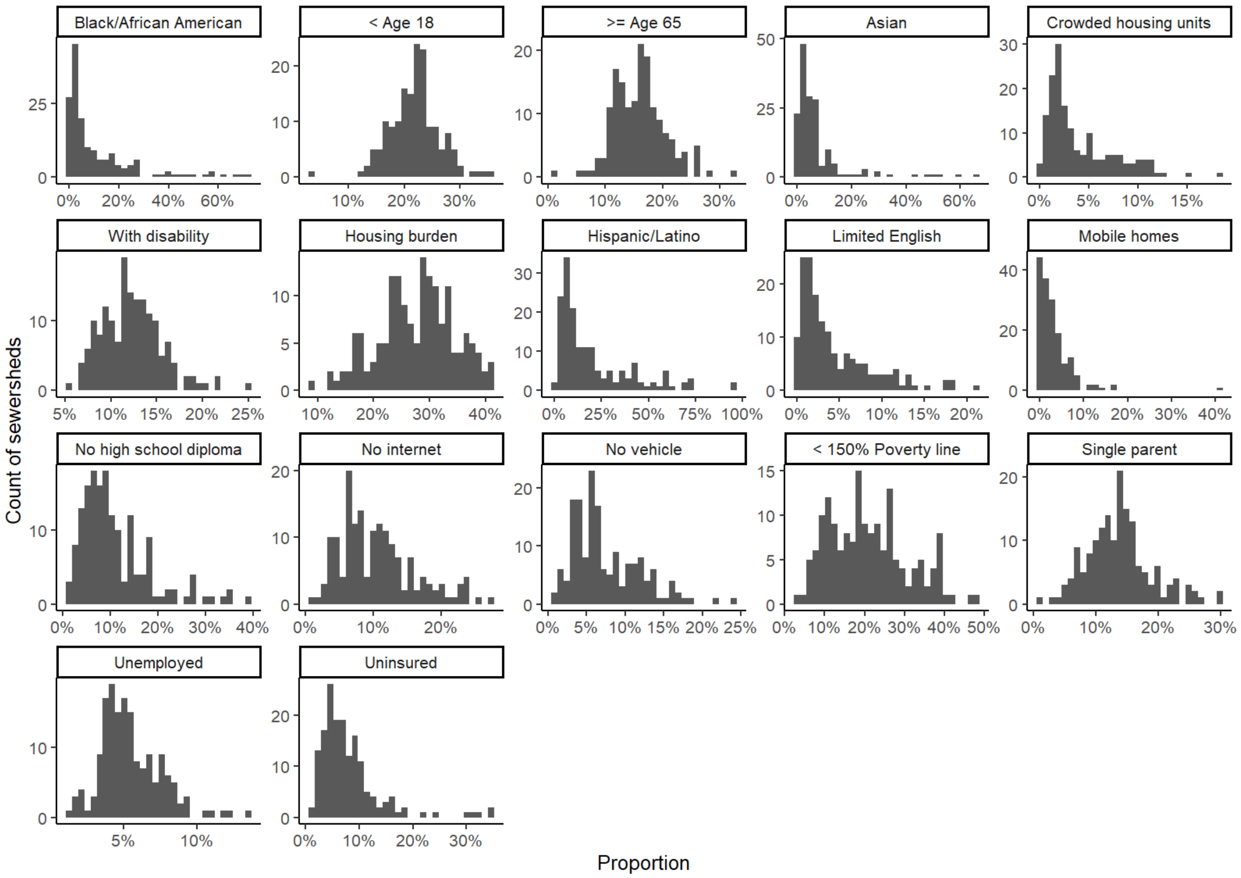

Figure S9. **Distribution of each variable in Table S4 among sewersheds.**

Urbanicity and population density. We used 2020 urban-rural designations from the US Census Bureau to determine the extent to which sewersheds are classified as urban.[15] For each sewershed, we used the Tabulate Intersection geoprocessing tool in ArcGIS Pro to determine the area proportion of the sewershed that intersects any urban area. We assigned a value of 0 if a sewershed did not intersect any urban area. The distribution of the urban proportion among sewersheds in this study is shown in **Figure S10**. To determine population density, we obtained the population of each county from the 2022 5-year ACS (subject table S0101) and the land area in square meters of each county from the 2022 US Census Bureau county boundaries (5-meter resolution).[13,14] We calculated the population density in square kilometers of each county by dividing the population by the land area and multiplying by 1000^2. We assigned the population density of each sewershed as the population density of its predominant county (i.e., the county that the majority of the sewershed is located in). We determined the predominant county of each sewershed using the Tabulate Intersection geoprocessing tool in ArcGIS Pro

and 2022 county boundaries (5-meter resolution) from the US Census Bureau.[14] The distribution of population density among sewersheds in this study is shown in **Figure S11**.

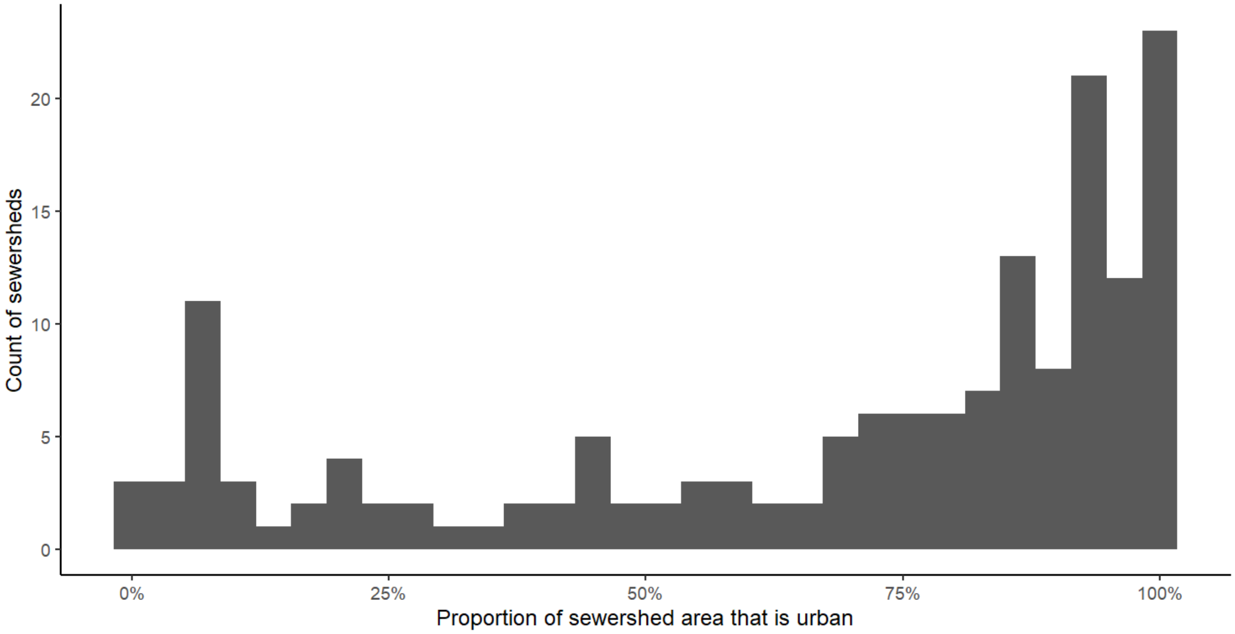

Figure S10. **Distribution of urbanicity among sewersheds.**

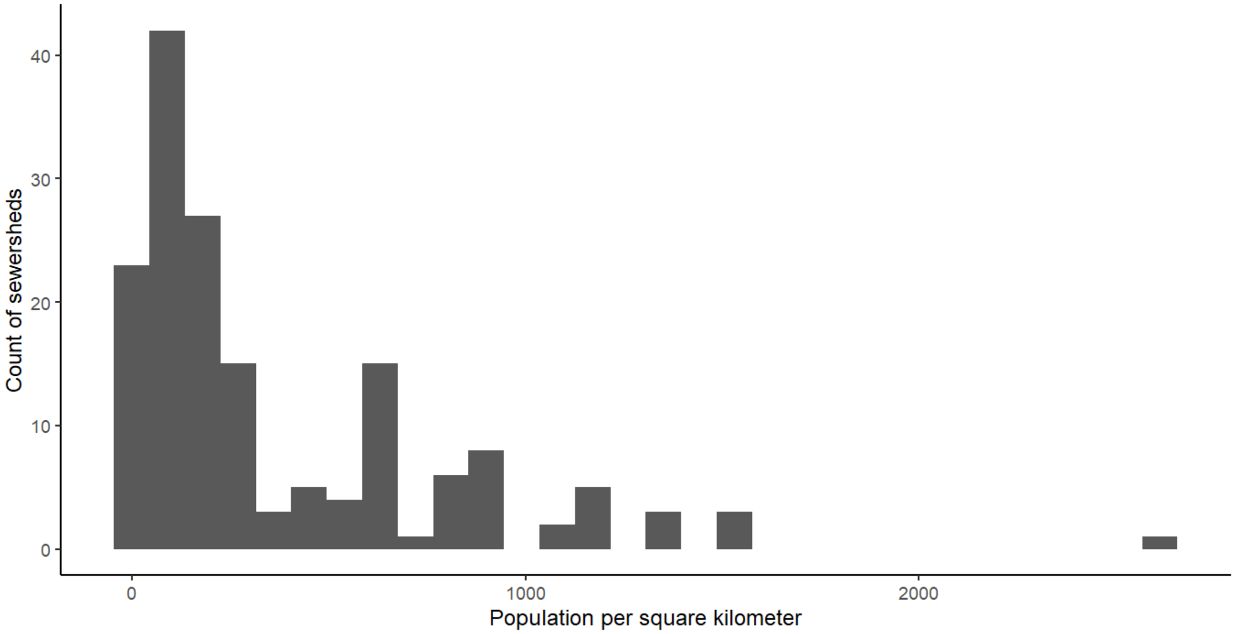

Figure S11. **Distribution of population density among sewersheds.**

Points of interest. We obtained the locations of major airports (defined as usage category "1,000,000 or more") from Esri (source: Federal Aviation Administration's National Airspace System Resource Aeronautical Data Product).[16] We obtained the locations of hospitals and nursing homes from the US Department of Homeland Security's Homeland Infrastructure Foundation-Level Data database.[17] We used the Tabulate Intersection geoprocessing tool in

ArcGIS Pro to determine the number of airports, hospitals, and nursing homes in each sewershed. For hospitals and nursing homes, we omitted any locations with a non-open status prior to using the Tabulate Intersection tool. **Figure S12** shows the distribution of airports, hospitals, and nursing homes among sewersheds in this study.

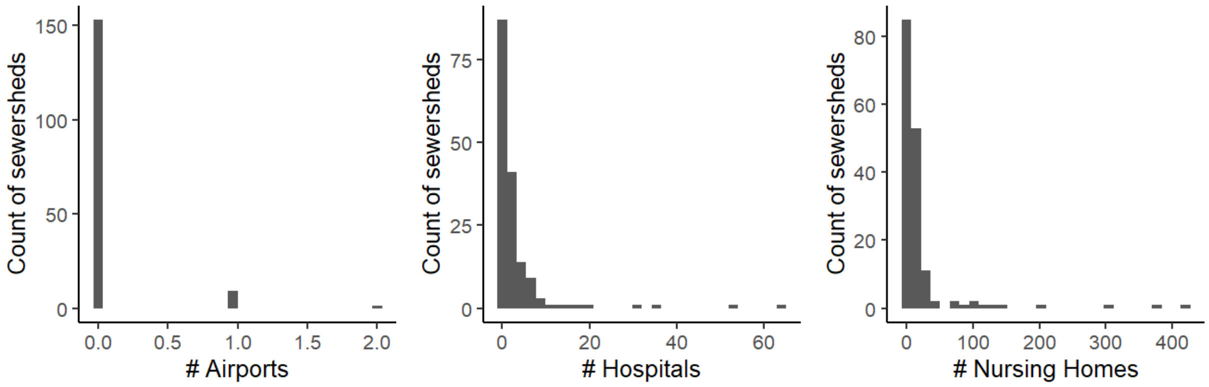

Figure S12. **Distribution of points of interest among sewersheds.**

Agricultural activity. Using the US Department of Agriculture (USDA) National Agricultural Statistics Service (NASS), we obtained 2022 inventory numbers for cattle, chickens, and hogs at the county resolution (**Table S5**).[18] For each sewershed, we assigned the inventory numbers of its predominant county (described above) to use as an indicator of agricultural activity in the sewershed. If inventory values were not reported or withheld to disclose numbers for individual operations, we assumed a value of zero. The Newark, NJ sewershed had no reported data for its predominant county, so we assigned inventory numbers from its second most predominant county to use for the analysis. The Census Bureau provides boundaries for county equivalents in Connecticut rather than counties which are used in the USDA NASS dataset. We assumed the Western Planning Region county equivalent is most similar to Fairfield county to determine the predominant county for the Stamford, CT sewershed. For chickens, we summed over all data items (broilers, layers, pullets, roosters). The distribution of cattle, chickens, and hogs among sewersheds in the study is shown in **Figure S13**.

Table S5. US Department of Agriculture National Agricultural Statistics Service Selections

**Program**	**Census**
Sector	Animals & Products
Group	Poultry; Livestock
Commodity	Cattle; Chickens; Hogs
Category	Inventory
Data Item	Cattle, Incl Calves - Inventory<br>Chickens, Broilers - Inventory<br>Chickens, Layers - Inventory<br>Chickens, Pullets, Replacement - Inventory

	Chickens, Roosters - Inventory<br>Hogs - Inventory
Domain	Total
Geographic Level	County
State	All
Year	2022
Period Type	Point in Time
Period	End of Dec

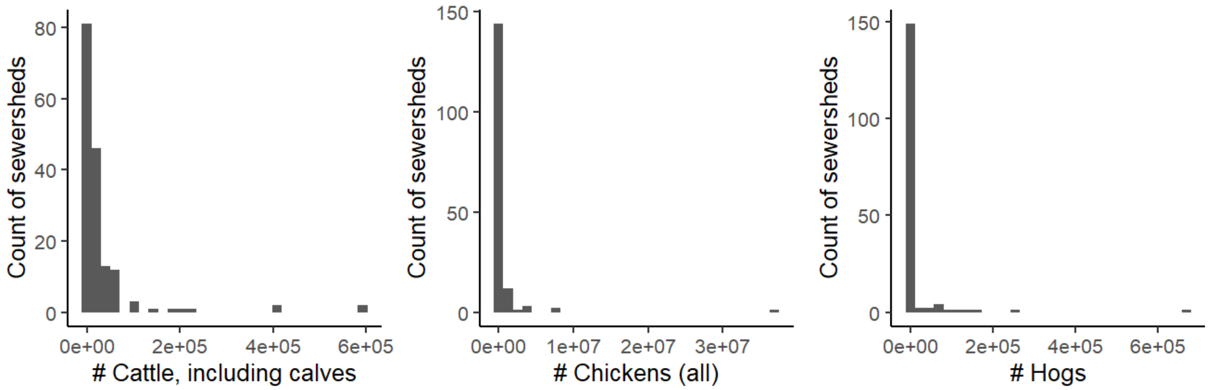

Figures S13. **Distribution of agriculture animal inventory numbers among sewersheds.**

Epic Cosmos data. All encounters in the Epic Cosmos dataset were temporally limited to encounters from June 1, 2023–June 1, 2024. Encounters were further geographically limited to the 116 counties representing the predominant county serviced by wastewater treatment plants (described above). Encounters were then filtered for all encounters with beta-lactam antibiotics (penicillins, cephalosporins, and carbapenems), tetracycline, vancomycin, and colistin antibiotics dispensed. The annual numbers of encounters with these prescriptions were then divided by the total number of encounters in the analysis period for each county. No colistin use was reported in the Epic dataset, so this variable was ultimately not included.

Table S6. Mean and standard deviation of the total number of copies of target per reaction for each target

**Target**	**n**	**mean**	**sd**
CMY	445	0.0451	0.0607
CTX-M	445	0.0175	0.0328
KPC	445	0.0766	0.118
mecA	444	0.000735	0.00112

NDM	445	0.0106	0.05
OXA-48	442	0.00733	0.0184
TEM	445	0.0844	0.102
VIM	445	0.128	0.214
mcr-1	442	0.000260	0.000474
tetW	445	0.183	0.169
vanA	445	0.00866	0.072
16S	439	0.321	0.215

Copies per target calculated by dividing the number of positive droplets by the number of accepted droplets. Note that mean and sd were calculated using all samples, not just those with detectable target.

Table S7. Statistics for Wilcoxon post-hoc test in Figure 2.

	Test statistics	Confidence interval	Effect size	Degrees of freedom	Adjusted p-values
Total burden - hospital	1345	(-1.4, -0.13)	0.20	133	0.045
Total burden - nursing	1596	(-1.4, -0.30)	0.25	133	0.020
Colistin burden - airport	262	(-0.73, -0.14)	0.24	153	0.004
Colistin burden - nursing	2201	(-0.16, -0.03)	0.22	153	0.007
Colistin burden - density	1134	(-0.44, -0.12)	0.33	153	0.0001
Colistin burden - urban	1352	(-0.22, -0.07)	0.34	153	0.0001
CMY - urban	1699	(-9.2e-5, -7.7e-6)	0.22	154	0.033
NDM - airport	354	(-2.6e-6, 8.1e-6)	0.22	160	0.006

NDM - hospital	1969	(-8.1e-6, 4.1e-6)	0.18	160	0.022
NDM - nursing	2138	(-6.4e-6, 8.1e-6)	0.29	160	0.0003
NDM - density	1329	(-4.1e-6, 8.1e-6)	0.32	160	0.0002
NDM - urban	1056	(-8.1e-6, 5.0e-7)	0.45	160	7.7e-8

Environmental Microbiology Minimum Information Checklist

Study Description

Study: [Study Name]
Date: 07-Jul-2021
Completed by: [Filled By...]

Environmental Sampling	Sample Treatment	Sample Reduction	Nucleic Acid Extraction	Reverse Transcription	PCR Detection	Analysis
Described in methods section	☐ Performed<br>No sample treatment performed	☐ Performed<br>Centrifugation was used, as described in the methods and referenced paper	Methods described briefly in pre-analytical processing and nucleic-acid extraction section and in references.	☐ Performed<br>Not performed	☐ qPCR ■ dPCR<br>Methods provided including the dMIQE checklist	QA/QC criteria and analysis described in methods

Control Checklist

	Environmental Sampling	Sample Treatment	Sample Reduction	Nucleic Acid Extraction	Reverse Transcription	PCR Detection
Step performed	■	☐	■	■	☐	■
**Negative Controls**						
Step has control info	☐	☐	☐	☐	☐	■
# control replicates	0	0	0	3	0	3
Control result reported	☐	☐	☐	☐	☐	■
Data handling reported	☐	☐	☐	☐	☐	■
**Positive Controls**						
Control introduced	☐	☐	☐	■	☐	■
Internal/External	N/A	N/A	Internal	External	N/A	External
Independent/Parallel	N/A	N/A	Parallel	Independent	N/A	Independent
Step has control info	☐	☐	☐	■	☐	■
# control replicates	0	0	0	1	0	1
Control result reported	☐	☐	☐	■	☐	■
Data Handling reported	☐	☐	☐	■	☐	■

Process Checklist

Environmental Sampling

- ■ Sampling Procedure
- ■ Number of samples
- ■ Sample amount, mean, range
- ■ Sampling locations, dates, times

Sample Treatment

- ☐ Performed
- ☐ Treatment procedure
- ☐ Reagents

Sample Reduction

- ■ Performed
- ■ Reduction procedure
- ■ Reagents
- ☐ Concentration Factor

Nucleic Acid Extraction

- ■ Extraction procedure
- ■ Amount extracted, amount obtained
- ■ Extract storage conditions

qPCR or dPCR

- ■ Target gene name, amplicon length
- ■ Thermocycling temperatures and times
- ■ Master mix: composition, vendors, concentrations
- ■ Additives: vendors, concentrations
- ■ Template amount added, pre-treatment (if any)
- ■ Primers: sequences, concentrations, vendors, references
- ■ Amplicon confirmation method (probe, melt curve, etc)
- ■ Probe sequence, concentration, vendor, reference
- ■ Instrumentation
- ☐ Equivalent volume of sample analyzed by PCR
- ■ Inhibition assessment procedure
- ■ Inhibition control description (if used)
- ■ Number samples tested and found inhibited

Reverse Transcription

- ☐ Performed
- ☐ One or two step
- ☐ cDNA storage conditions (if two step)
- ☐ Reaction temperatures and times
- ☐ Reaction reagents and concentrations
- ☐ Priming method
- ☐ Reaction volume, added template amount
- ☐ Inhibition assessment procedure
- ☐ Inhibition control description (if used)
- ☐ Number samples tested and found inhibited

Analysis – dPCR

- ■ Threshold settings
- ■ Technical replicates, number, well merging
- ■ Partitions measured, number, mean, variance
- ■ Partition volume
- ■ Target copies per partition, mean, variance
- ■ Program used for dPCR analysis
- ■ Explanation of control results, example plots

Analysis – qPCR

- ☐ Method for handling failed negative controls
- ☐ Technical replicates, number, calculations
- ☐ Calibration standards: description and source
- ☐ Method of quantifying standards
- ☐ Calibration curve slope
- ☐ Calibration curve R2
- ☐ Lowest standard measured or 95% LOD
- ☐ Cq value determination method

References

1. Schmidt, G. V. *et al.* Sampling and Pooling Methods for Capturing Herd Level Antibiotic Resistance in Swine Feces using qPCR and CFU Approaches. *PLOS ONE* **10**, e0131672 (2015).
2. Pholwat, S. *et al.* Genotypic antimicrobial resistance assays for use on E. coli isolates and stool specimens. *PLOS ONE* **14**, e0216747 (2019).
3. Garcia, L. S. *Clinical Microbiology Procedures Handbook, 3rd Ed.* (ASM Press, 2010).
4. Böckelmann, U. *et al.* Quantitative PCR Monitoring of Antibiotic Resistance Genes and Bacterial Pathogens in Three European Artificial Groundwater Recharge Systems. *Appl. Environ. Microbiol.* **75**, 154–163 (2009).
5. Chavda, K. D. *et al.* Evaluation of a Multiplex PCR Assay To Rapidly Detect Enterobacteriaceae with a Broad Range of β-Lactamases Directly from Perianal Swabs. *Antimicrob. Agents Chemother.* **60**, 6957–6961 (2016).
6. Lutgring, J. D. *et al.* Phenotypic and Genotypic Characterization of *Enterobacteriaceae* Producing Oxacillinase-48–Like Carbapenemases, United States. *Emerg. Infect. Dis.* **24**, 700–709 (2018).
7. Roschanski, N., Fischer, J., Guerra, B. & Roesler, U. Development of a Multiplex Real-Time PCR for the Rapid Detection of the Predominant Beta-Lactamase Genes CTX-M, SHV, TEM and CIT-Type AmpCs in Enterobacteriaceae. *PLoS ONE* **9**, e100956 (2014).
8. Yang, D. *et al.* The Occurrence of the Colistin Resistance Gene mcr-1 in the Haihe River (China). *Int. J. Environ. Res. Public. Health* **14**, 576 (2017).
9. Smith, M. S. *et al.* Quantification of Tetracycline Resistance Genes in Feedlot Lagoons by Real-Time PCR. *Appl. Environ. Microbiol.* **70**, 7372–7377 (2004).
10. Farivar, T. N. *et al.* Development and evaluation of a Quadruplex Taq Man real-time PCR assay for simultaneous detection of clinical isolates of Enterococcus faecalis, Enterococcus

faecium and their vanA and vanB genotypes. *Iran. J. Microbiol.* **6**, 335–340 (2014).

11. Suzuki, M. T., Taylor, L. T. & DeLong, E. F. Quantitative Analysis of Small-Subunit rRNA Genes in Mixed Microbial Populations via 5′-Nuclease Assays. *Appl. Environ. Microbiol.* **66**, 4605–4614 (2000).
12. U.S. Centers for Disease Control and Prevention. Social Vulnerability Index 2022. https://www.atsdr.cdc.gov/place-health/php/svi/svi-data-documentation-download.html?CDC_AAref_Val=https://www.atsdr.cdc.gov/placeandhealth/svi/data_documentation_download.html.
13. U.S. Census Bureau. 2022 5-Year American Community Survey (ACS). https://www.census.gov/programs-surveys/acs.
14. U.S. Census Bureau. Cartographic Boundary Files. https://www.census.gov/geographies/mapping-files/time-series/geo/cartographic-boundary.html.
15. U.S. Census Bureau. 2020 Census Urban-Rural Classification. https://www.census.gov/programs-surveys/geography/guidance/geo-areas/urban-rural.html (2024).
16. Esri Data and Maps. USA Airports 1,000,000 or more. https://hub.arcgis.com/datasets/esri::usa-airports/about?layer=1.
17. US Department of Homeland Security. Homeland Infrastructure Foundation-Level Data. https://hifld-geoplatform.hub.arcgis.com/ (2024).
18. U.S. Department of Agriculture. 2022 National Agricultural Statistics Service Quick Stats. https://quickstats.nass.usda.gov/.
